# Human nasal rhinosporidiosis: an Italian case report

**DOI:** 10.1186/1746-1596-1-25

**Published:** 2006-08-31

**Authors:** Luca Morelli, Mario Polce, Francesco Piscioli, Franca Del Nonno, Renato Covello, Alessia Brenna, Antonio Cione, Stefano Licci

**Affiliations:** 1 Division of Pathology, "S. Maria del Carmine" Hospital, Rovereto, Italy; 2 Division of Otorhinolaryngology, "S. Maria del Carmine" Hospital, Rovereto, Italy; 3 Division of Pathology, National Institute for Infectious Diseases "L. Spallanzani", Roma, Italy; 4 Division of Pathology, Istituto Regina Elena per lo studio e la cura dei tumori, Roma, Italy

## Abstract

**Background:**

Rhinosporidiosis is a disease affecting primarily the mucosa of nose, conjunctiva and urethra. It is endemic in some Asiatic regions, affecting people of any age and sex. Its manifestation is a polypoid mass growing inside the affected cavity and the only treatment is surgical excision. *Rhinosporidium seeberi *is the aetiological agent. Many discussions arouse regarding the taxonomic classification of the microorganism, recent studies established it is an aquatic protistan parasite. The lesion may recur and sometimes cause osteolytic bone lesions. In endemic areas it is not easy to establish if recurrent lesions are due to relapse or reinfection.

**Case presentation:**

A 26-year-old male patient from India, resident in Italy since 2005, presented in March 2006 with a history of nasal obstruction of three months duration. Physical examination showed an erythematous, papillomatous mass, 3 cm in diameter, obstructing the right nasal cavity. A microscopic diagnosis of rhinosporidiosis was made. Few Italian human cases of this disease have been previously reported in the literature.

**Conclusion:**

Rhinosporidiosis is a condition which both clinicians and pathologists should keep in mind when managing patients from endemic countries with nasal masses. Moreover, it is very interesting in such cases to follow the clinical course: an eventual recurrence of the lesion in our patient would mean a true relapse, excluding the possibility of a reinfection, more probable in the endemic areas.

## Background

Rhinosporidiosis is an infectious disease, endemic in some areas of Asia, such as India and Ceylon, although it may occur in the Americas, Europe, Africa, more commonly in the tropics. Outbreaks of human and animal disease have been recently described in Europe and reports of sporadical cases in temperate and tropical countries continue to appear [1,2]. The aetiological agent is *Rhinosporidium seeberi*, whose taxonomy has been debated in the last decades [3], since the microrganism is intractable to isolation and microbiological culture. Moreover, it shows morphological features resembling those of fungi and protozoa [4]. The biological agent has a mature stage that consists of large, thick-walled spherical structures (called sporangia) containing smaller "daughter cells" (called "sporangiospores"), and it can be visualized with fungal stains as Gomori methenamine silver (GMS) and periodic acid-Schiff (PAS), as well as with standard haematoxylin and eosin (H&E) staining. Clinically, the lesion presents as a polypoid, soft mass, sometimes pedunculated, of the nose (primary site of infection), the eye and its adnexa, above all conjunctiva, or the urethra. Larynx, trachea, skin and lung are less frequently involved. Osteolytic bone infiltration is another clinical presentation. Generalized rhinosporidiosis with skin and visceral involvement is extremely rare [5]. The only curative approach is the surgical excision combined with electrocoagulation. There is no demonstrated efficacy in using antifungal and/or antimicrobial drugs. Recurrence, dissemination in anatomical close sites and local secondary bacterial infections are the most frequent complications. Few cases have been previously described in Italy: two native Italian cases were reported by Karunaratne in 1963 (cited trough van der Coer review about rhinosporidiosis in Europe in 1992) [6]. Two more case were described by Di Tondo et al. in 1985 and by Gori and Scasso in 1994 [7,8].

## Case presentation

### Clinical history

A 26-year-old male patient from India, resident in Italy since 2005, presented in March 2006 to the Department of Otorhinolaryngology with a history of nasal obstruction of three months duration. The family history of the patient revealed a similar disease in his mother, which recurred twice (in 1994 and in 2000). The patient referred recurrent epistaxis since the age of 7, treated with cold packs, and a not better specified conjunctival inflammation cured with eyedrops in September 2005. Physical examination showed an erythematous, papillomatous mass, 3 cm in diameter, obstructing the right nasal cavity, attached by a narrow pedicle to the nasal septum. No abnormality was seen in the controlateral nasal cavity or nasopharynx. The mass was resected endoscopically and the base of implant was electrocoagulated. The patient did not assume any drug therapy and, until today, after five months of follow-up, during which he underwent a clinical exam twice, he is healthy with no sign of recurrence.

### Macroscopic and microscopic findings

**Figure 1:**
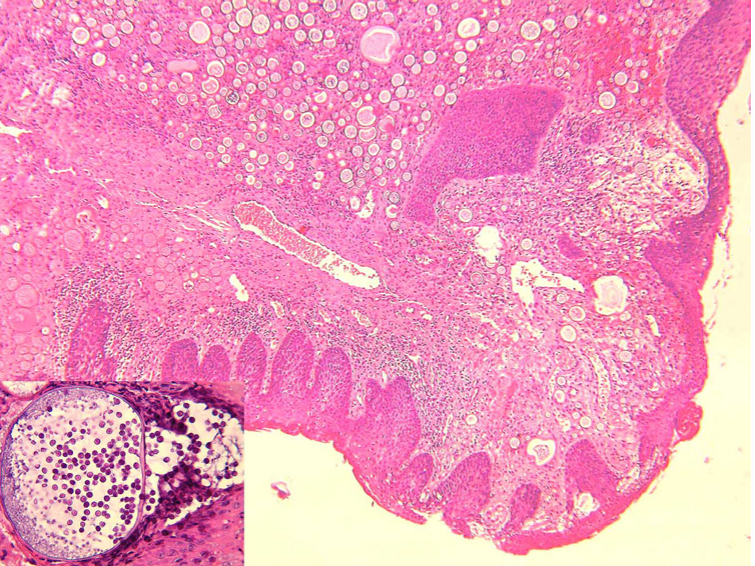
Nasal rhinosporidiosis. In the insert, globular cyst containing endospores (Haematoxylin & Eosin, 50×; in the insert, 400×)

**Figure 2:**
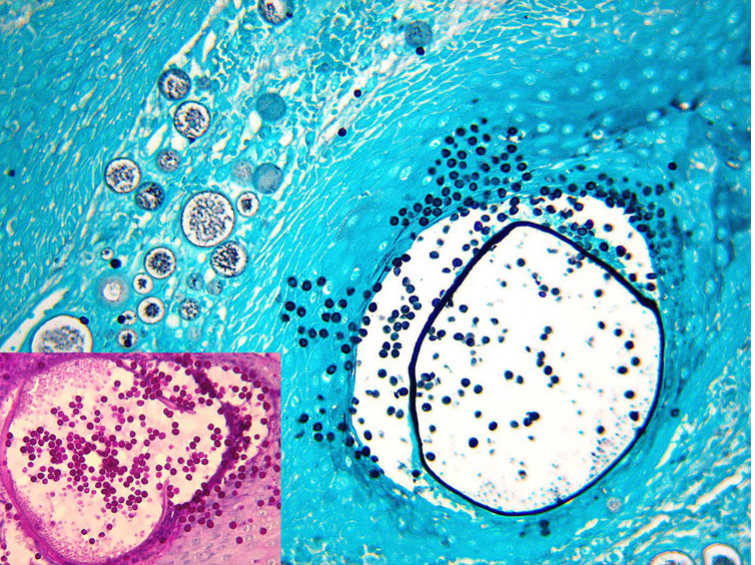
Gomori methenamine silver stain and, in the insert, periodic acid-Schiff stain (200×; in the insert, 400×).

The excised mass weighed 4 grams, measured a maximum diameter of 3 cm, was pink, with a fleshy consistence, studded with whitish spots on its surface. On histological examination, the lesion showed the characteristic features of the rhinosporidiosis: the polypoid fibroconnective stroma, covered by flat multi-stratified squamous epithelium, contained many globular cysts. Each of these cysts represented a thick-walled sporangium containing numerous "daughter spores" in different stages of development (Fig. 1). The stroma contained a vascular fibroconnective tissue with fibroblasts and myofibroblasts and an inflammatory infiltrate (neutrophil granulocytes, lymphocytes, plasma cells and histiocytes). Histochemical stains such as PAS, GMS (Fig. 2) and mucicarmine were used to establish the correct diagnosis of rhinosporidiosis. Morphological criteria were based on the diameter of the endospores and sporangia, respectively 5–10 μm and 50–1000 μm. These findings made easier the distinction of *Rhinosporidium seeberi *from another common nasal mycosis aetiological agent, *Coccidioides immitis*.

## Conclusion

Rhinosporidiosis is a rare chronic granulomatous disease, endemic in South India, Sri Lanka and some areas of the African continent. Infrequently, isolated cases were reported in other parts of the world, mainly due to the socio-cultural phenomenon of the migration. The major debate about the aetiological agent, *Rhinosporidium seeberi*, is regarding its precise taxonomy. Most microbiologists initially considered it a fungus just because of its property to be stained by fungal stains such as GMS and PAS; the uncertainty arises from the difficulty to isolate and to grow the microrganism in culture (only in tissue cultures some could grow it through an entire life cycle). Some authors recently postulated that the aetiological agent of the disease was not a fungus but a prokaryotic cyanobacterium called *Microcystis aeruginosa*. This hypothesis was based on the finding of this bacterium in rivers and ponds where patients with rhinosporidiosis used to bathe, and supported by laser-scanning confocal, light, electron microscopy and molecular findings [9-12]. Nevertheless, this observation can not provide a compelling evidence. Moreover, some authors found no evidence of a relationship between this microrganism and *Rhinosporidium seeberi *[12], whereas others went to the conclusion (the most accepted today) that the responsible agent is an aquatic protistan parasite belonging to a novel group of fish parasites (Mesomycetozoa), located phylogenetically between the fungal and animal divergence [4,13].

*Rhinosporidium seeberi *should be distinguished from another microrganism, *Coccidioides immitis*. This latter has similar mature stages represented by large, thick-walled, spherical structures containing endospores, but the spherules are smaller (diameter of 20–80 μm versus 50–1000 μm) and contain small endospores (diameter of 2–4 μm). Moreover, Coccidiodes does not stain with the mucicarmine.

The pathway of transmission of Rhinosporidium remains unclear, but, since the most affected sites are the nose and the eye, it has been suggested that it is air- or water-borne; water and soil are believed to be the reservoir of infection, given the high incidence of the disease in sandworkers, paddy cultivators and people bathing in stagnant waters. It is interesting in our case that the patient treated his chronic epistaxis only with cold packs: it is likely that in his country people used to make cold packs with contaminated water; thus, many cases of recurrence could be interpreted as reinfections, and this could explain the occurrence of the same disease in his mother for two times (in 1994 and in 2000). The "conjunctive inflammation" he reported could be related to the same infection too, but we do not have sufficient clinical elements to debate about that. An important feature of the life cycle of the Rhinosporidium that we could argue is its prolonged incubation before clinical manifestation: we can presume that the patient was infected before his arrival in Italy, and the disease manifested as a polypoid nasal mass at least after one year; indeed it is very likely that the real first exposition to the aetiological agent happened many years ago, when the patient was a child; recurrent epistaxis, one of the most frequent initial clinical signs, presented since the age of 7, i.e. 19 years ago.

At present, the treatment for rhinosporidiosis is the surgical excision. Some authors proposed a medical therapy with dapsone [14,15], but the results are not convincent. Antimicrobial therapy is ineffective, and the disease may recur after months or years.

In conclusion, this is an infrequent Italian human case of rhinosporidiosis, rare cases being previously reported in the literature. Rhinosporidiosis is a condition which both clinicians and pathologists should keep in mind when managing patients from endemic countries with nasal masses. Moreover, it will be very interesting to follow in the next years the clinical course of our patient: an eventual recurrence of the lesion, usually occurring after a long time, would mean a true relapse, excluding the possibility of a reinfection, more probable in the endemic areas.

## Competing interests

The author(s) declare that they have no competing interests.

## Authors' contributions

AB and AC carried out routine and special histochemical stains. LM, MP, FP, FDN, RC and SL participated equally in the design of the report and in drafting the manuscript. All authors read and approved the final manuscript.
